# Body mass index and mortality in a nationally representative cohort of south African adults

**DOI:** 10.1016/j.gloepi.2025.100220

**Published:** 2025-09-26

**Authors:** Annibale Cois

**Affiliations:** Burden of Disease Research Unit, South African Medical Research Council, Francie van Zijl Drive, Parow Valley 7500, Cape Town, South Africa & Division of Epidemiology & Biostatistics, School of Public Health, University of Cape Town, South Africa.

**Keywords:** Body mass index, Obesity, All-cause mortality, South Africa, Survival analysis

## Abstract

**Aim:**

To examine the association between Body Mass Index (BMI) and all-cause mortality in South Africa.

**Methods:**

Longitudinal data on adults 20 years and older from five waves (2008, 2010–11, 2012, 2014–15, and 2017) of the South African National Income Dynamics Study were analysed. Survival proportional hazard models, adjusted for sociodemographic and lifestyle characteristics, were used to estimate the relationship between BMI and mortality. Sensitivity analyses were conducted to assess the robustness of the estimates.

**Results:**

Of the 12,402 eligible individuals, 10917 had valid BMI measurements and were included in the analyses. During a total of 83,077 person-years of observation, 1741 individuals died.

Hazard ratios for all-cause mortality were significantly lower in the BMI range 25–40 kg/m^2^ in comparison with the reference category of 18.5–25 kg/m^2^ and were minimal in the range 30–35 kg/m^2^ (HR = 0.68, 95% CI: 0.50–0.88). BMI < 18.5 kg/m^2^ was associated with an increased risk of death, with a maximum hazard ratio of 2.14 (95% CI: 1.36–3.4) in the <16 kg/m^2^ category. The pattern was repeated in the sex-specific analyses. The relationship persisted after restricting the analyses to never smokers, excluding subjects with pre-existing conditions or who died in the first two years of follow-up.

**Conclusions:**

This study suggests that, in the South African adult population, BMI in the overweight or mild obesity range according to international definitions is associated with a reduced risk of mortality compared to the” healthy weight” range. Further research is needed to corroborate these results.

## Introduction

Body mass index (BMI) is widely used as an indicator of adiposity, and numerous studies have documented a U-shaped relationship between BMI and all-cause mortality, with both low and high values associated with an increased risk of premature death [[1], [2], [3], [4], [5], [6], [7], [8], [9]].

At the extremes of the BMI distribution, peculiar morbidity patterns have been identified, supporting the biological plausibility of the observed heightened mortality. Low values of BMI are associated with impaired immune function, higher susceptibility to infections, reduced muscle function, skeletal disorders, anaemia, delayed wound healing and recovery from surgery, and accelerated decline in cognitive function and functional status among the elderly [[10], [11], [12]]. At the other extreme, high BMI values are strong predictors of the development of several chronic diseases, including type two diabetes, hypertension, coronary artery disease, stroke and some types of cancer [[13], [14], [15], [16]].

However, although the majority of primary studies and the results of large meta-analyses involving millions of individual data points agree on the existence of increased mortality risk at the extreme of the BMI distribution [1,2,7], the actual shape of the relationship between BMI and mortality across populations with diverse characteristics remains controversial, with evidence suggesting that demographic, socioeconomic and ethnic differences influence the strength and nature of this association. Studies have often found that the association between BMI and mortality is stronger for men, tends to weaken with age, and the BMI range corresponding to minimum mortality risk increases in older ages [[17], [18], [19], [20]]. Socioeconomic status (SES) can also modify the relationship, which becomes more pronounced among individuals with low SES, especially men [21,22]. The evidence of different relationships across ethnic groups has also been repeatedly documented, and it is generally accepted that South Asian populations experience heightened mortality risk at lower BMI compared to other ethnic groups, while the opposite has been observed, at least in the US, for Black populations compared to their White counterpart [18,[23], [24], [25], [26], [27]].

Understanding these differences and the extent to which the overall relationships – estimated mostly based on data collected in high-income countries (HICs) with reliable surveillance and vital registration systems in place – are applicable in the profoundly different contexts of low- and middle-income countries (LMICs) is crucial for refining public health recommendations and tailoring interventions to reduce mortality risk related to sub-optimal body weight in a way that accounts for their distinct socioeconomic and health profiles.

Sub-Saharan African countries represent a case in point, where this need is especially evident, for various reasons. First, these countries are characterised by demographic and ethnic makeup, socioeconomic and environmental contexts, health systems and disease burdens largely different from those where data underlying current estimates of the BMI-mortality relationship have been collected. Considering the observed variation of this relationship across demographic, socioeconomic and ethnic strata briefly reviewed above, it is plausible that this relationship might differ in these settings. Second, LMICs populations are experiencing demographic, epidemiologic and behavioural transitions (comprising, the latter, changes in traditional diet and levels of physical activity) which include a significant right-shift of the population distributions of BMI. Understanding the potential consequences of this transformation in terms of future mortality and burden of disease is, consequently, of extreme interest in a public health and health system perspective. Third, plenty of literature has highlighted ethnic differences and strong cultural influences on body image perception, together with complex phenomena of stigmatization often acting in opposite directions across sociocultural contexts [[28], [29], [30], [31]]. Unlike typical Westernised populations, in many African contexts, ‘thinness’ has a strong negative connotation for its association with disease and lack of resources. The health consequences of imposing ‘imported’ – and potentially unjustified – standards of ‘ideal’ body weight that conflict with community and personal views must also be taken into account in the planning of public health activities [[32], [33], [34], [35], [36]].

Despite increasing awareness of these factors, however, studies on the link between BMI and mortality in African populations remain lacking. None of the large meta-analyses cited in support of the U-shaped relationship between BMI and all-cause mortality includes studies from the African continent [1,2,4,9]. To the author's knowledge, only one recent study in South Africa has directly addressed the estimation of this relationship from longitudinal data [37].

This study aims to reduce this knowledge gap by analysing the relationship between BMI and all-cause mortality in the adult population of South Africa, a middle-income sub-Saharan country in full demographic and epidemiological transition. The study uses longitudinal data on a nationally representative sample of the South African population with baseline data collection in 2008 and 10-year follow-up.

## Methods

### Data sources

Data collected within the scope of the South African National Income Dynamics Study (NIDS), a nationally representative panel survey of South Africa's residents, were used for this study [38]. The baseline data collection was conducted in 2008, when a two-stage cluster sample design was used to randomly select about 7300 households across 400 primary sampling units, stratified by district council (a second level administrative division of South Africa's territory into 52 areas). All available individuals 15 years and older in the selected households were included in the data collection. The consenting individuals were recontacted in 2010–2011, 2012, 2014–2015 and 2017. Details on the strategy and realization of the NIDS sample are provided in the survey technical materials [39,40]. These include methods of calculation and calibration of the sampling weights provided with the datasets to take into account the sampling design and the unequal response rates across population strata [41].

This study only considers the subsample of non-pregnant individuals aged 20 years and older at baseline.

### Data collection

Data collection was realised by the NIDS research team. Information on demographic characteristics and lifestyle risk factors was collected using a standard questionnaire administered by trained staff. Racial ascription was self-defined by participants according to the historical “population group” classification used in South Africa during apartheid and retained in the official statistics: Asian (mainly Indian descent), Black (or African), Coloured (wide grouping of people of mixed ancestry) and White (mainly European descent) [42]. Current or past smoking, alcohol consumption (current regular, current occasional, past), frequency of physical exercise (never, less than once a week, twice a week, three or more times a week), and previous diagnoses of chronic conditions by a health professional (tuberculosis, asthma, hypertension, diabetes, stroke, heart diseases, cancers) were self-reported by participants in response to direct questions. Duplicate measures of weight and height were recorded, with a third measure taken if their difference was greater than 0.5 kg or 0.5 cm respectively.

The vital status of the cohort members was assessed at each wave of data collection, based on reports of deaths by other household members. The cause of death was only recorded as ‘natural’ or ‘accidental’. The date of death was recorded exactly when available, otherwise participants were assumed to have died at an unspecified date between the last time they were recorded as alive and the date when first reported as dead.

Details on the tools and procedures are available in the NIDS website (http://www.nids.uct.ac.za/nids-data/documentation).

### Data preprocessing

Participants not interviewed and not confirmed dead by 2017 were classified as lost to follow-up (LTFU) at the date of the last interview or last time reported as alive in the household interview [44]. As a subsample of individuals interviewed at wave 5 of the NIDS survey were re-contacted in 2020/2021 for a derived study (NIDS CRAM survey, https://cramsurvey.org/), their identifiers were used for a partial validation of the vital status of subjects categorised as LTFU in the dataset used for this study, but this exercise did not result in changes to the original classification.

Education was categorised as Primary, Secondary, Tertiary and None according to years of completed schooling. The geographic type of residence was categorised as urban/rural according to Statistics South Africa's Census 2001 [43].

Excluding measures with implausible values (height < 130 cm or > 230 cm for males and < 110 cm or > 210 cm for females; weight < 35 kg or > 300 kg for males and < 25 kg or > 300 kg for females), the average of the remaining readings was considered as the subject's true value and used to calculate BMI in kg/m^2^. After excluding individuals whose BMI was missing or assumed implausible values (BMI < 10 kg/m^2^ or > 70 Kg/m^2^), the BMI of the remaining participants was classified into 7 categories, corresponding to the World Health Organization (WHO) extended BMI categories of *severe thinness* (BMI <16 kg/m^2^), *Moderate/Mild thinness* (16 ≤ BMI < 18.5), *Normal Weight* (18.5 ≤ BMI < 25), *Overweight* (25 ≤ BMI < 30), *Obesity Class I* (30 ≤ BMI < 35), *Obesity Class II* (35 ≤ BMI < 40), *Obesity Class III* (BMI ≥ 40) [46].

### Statistical analyses

Statistical analyses were conducted using R statistical environment v. 4.3.3 [45].

Baseline characteristics of participants were described as median and interquartile ranges (IQR) for continuous variables and frequency for categorical measures. Student *t*-test or Mann–Whitney *U* test (for continuous variables) and Χ^2^ test or Fishers' Exact test (for categorical variables) were used to investigate differences between participants with and without valid BMI measurements and between those with known vital status and those LTFU.

Person-time of follow-up was calculated from the date of the baseline data collection until the date of death, LTFU or the last household interview in 2017. Age-standardized mortality was calculated with the direct method using the five-year age-specific mortality and the age distribution of the South African population in 2008 [47].

A parametric proportional hazard survival model for censored outcomes as implemented in the R package *icenReg* [48] was used to estimate hazard ratios (HR) for the effect of BMI at baseline on survival. The model took explicitly into account the interval-censored nature of the survival data, given that in most cases the date of death or LTFU was only known as an interval. The assumption regarding the shape of the baseline distribution was tested by comparing the survival time distribution assumed by the model (Weibull) with the baseline distribution recovered non-parametrically by fitting an analogous Cox model. The plausibility of the proportional hazard assumption was tested graphically by comparing the shapes of the survival function across different BMI categories. The R^2^ statistic proposed by Royston was calculated to compare the proportion of variation explained across models [49].

The main model was adjusted for categorical age, sex, population group, household income quintile, marital status, frequency of physical exercise, smoking and alcohol use and their interaction, and seasonality (periodic spline with 3 internal knots). Cases with missing data in any of the covariates were excluded in the estimation of the relevant model. The BMI range 18.5–25 kg/m^2^ – the ‘healthy weight’ category according to major international organization including the WHO [50] – was used as the reference BMI category to present the modelling results. Sex and age differences in the BMI-mortality associations were assessed by refitting the model separately for males and females, and by age group (< 40 years, 40 to 59 years and 60 years and over).

Sensitivity analyses to examine the potential impact of reverse causality (i.e. confounding by weight loss caused by pre-existing disease) were conducted (1) by excluding participants with pre-existing chronic diseases and (2) by excluding participants who died in the first 2 years of follow-up. A similar analysis was conducted to address confounding by smoking, which is a known cause of weight loss and a risk factor for death. As previous studies suggest a different and potentially inverse relationship between BMI and mortality for external causes of death, the analyses were also restricted to participants who died from natural causes [7,51].

To assess the potential bias introduced by LTFU and by the exclusion of individuals with missing covariate values, the main model was re-estimated (1) by excluding individuals LTFU, (2) by inverse probability of censoring weighting (IPCW) and by preliminary multiple-imputation of missing values [52].

To better illustrate the potential public health relevance of the study results, the model was used to predict the absolute risk of dying in the following 5, 10 and 15 years for a population with the covariate distribution as the population under study but with different values of BMI at baseline.

All analyses took into account the complex sampling strategy of the NIDS survey, by incorporating sampling weights and calculating standard errors and confidence intervals adjusted for clustering and stratification by bootstrap (R package *surveybootstrap* [53]).

Further details on the statistical procedures and the R code used for the analysis are available as Supplementary Material 1 and 2.

### Ethics

The NIDS study has been granted ethics approval by the Commerce Faculty Ethics Committee at the University of Cape Town. The secondary analyses presented here were granted ethics exemption from the Stellenbosch University Health Research Ethics Committee (HREC Reference No: X21/07/017).

## Results

### Sample characteristics

Of the 12,402 eligible individuals interviewed at baseline, 1485 (12 %) were excluded because of missing or implausible BMI. The remaining *N* = 10,917 were observed for a total of 83,077 person-years, during which 1741 deaths were recorded, and 2257 participants (21%) were LTFU. A diagram describing the selection process and the evolution of the cohort is presented in Supplementary Fig. S3.

The sample included more women (61%) than men, with a median age at baseline of 41 years for the former and 38 years for the latter. The median BMI was 27.6 kg/m^2^ (range: 11.5–69.4 kg/m^2^) among females and 22.6 kg/m^2^ among males (11.6–65.2 kg/m^2^). The majority of participants were either unemployed (20%) or not actively looking for a job (35 %) and had primary or secondary education (71%). About a quarter were smokers (24 %) and either occasional or regular drinkers (27%). Almost three quarters (74%) never exercised. Hypertension was the most reported chronic disease (19% of participants), followed by tuberculosis (5.4%), diabetes (4.7%), asthma (3.9%), heart conditions (3.6%), stroke (1.1%) and cancer (0.7%). Further details on the baseline characteristics of the sample are reported in Table 1, separately by BMI categories.

**Table 1:** Baseline characteristics of the study sample according to Body Mass Index.

	Body Mass Index [kg/m^2^]								
	All sample	< 16	[16,18.5)	[18.5,25)	[25,30)	[30,35)	[35,40)	≥ 40	p
**N**	10,917	168	633	4506	2610	1634	817	549	
**Sex [%]**									
Female	60.7	41.7	39.0	46.0	65.8	79.4	89.1	90.0	< 0.001
**Age [Median (IQR), years]**	40.0 (28.0–54.0)	45.5 (28.0–54.2)	37.0 (26.0–53.0)	34.0 (25.0–49.0)	41.0 (30.0–55.0)	45.0 (34.0–57.0)	46.0 (36.0–56.0)	47.0 (37.0–58.0)	< 0.001
**Population group [%]**									< 0.001
Black African	78.9	68.5	73.3	81.8	78.0	73.7	80.9	81.6	
Coloured	14.2	28.6	24.8	13.1	12.2	16.0	12.9	13.5	
White	5.5	2.4	0.8	3.8	8.2	8.4	5.8	22 4.0	
Asian	1.3	0.6	1.1	1.3	1.6	1.8	0.5	5 0.9	
**Marital status [%]**									
Single	42.1	46.1	52.1	52.3	35.8	29.9	30.0	30.3	< 0.001
Married/Informal Union	44.3	35.9	37.4	37.7	49.8	52.5	50.9	48.5	
Widowed/Separated/Divorced	13.6	18.0	10.5	10.0	14.4	17.6	19.2	21.2	
Missing (N)	31	1	5	11	5	5	3	1	
**Education [%]**									
No school	18.8	25.6	23.9	18.2	19.0	18.5	15.2	20.7	< 0.001
Primary	31.0	45.5	36.6	29.9	28.1	30.7	35.5	36.3	
Secondary	39.9	25.6	34.5	42.9	40.1	38.1	38.5	33.5	
Tertiary	10.3	3.2	5.0	8.9	12.9	12.7	10.8	9.6	
Missing (N)	1485	12	76	679	362	214	95	47	
**Employment [%]**									
Not Economically Active	34.5	53.9	40.3	32.0	32.9	34.9	38.4	42.4	< 0.001
Unemployed	20.4	13.9	23.4	22.3	19.4	18.8	18.7	15.3	
Employed	45.1	32.1	36.3	45.7	47.7	46.3	42.9	42.4	
Missing [N]	97	3	10	37	21	18	2	6	
**Income Quintile [%]**									
I (poorest)	18.4	22.6	21.2	20.4	16.6	15.9	14.6	19.1	< 0.001
II	19.3	21.4	22.4	20.0	18.9	17.7	18.7	17.1	
III	20.6	25.0	24.2	20.7	18.4	19.8	23.3	24.2	
IV	21.3	23.8	22.7	22.2	20.3	19.8	20.8	21.5	
V (richest)	20.4	7.1	9.5	16.8	25.7	26.8	22.6	18.0	
**Geotype [%]**									
Rural Formal	11.6	14.3	14.5	13.2	11.7	9.7	6.5	7.7	< 0.001
Tribal Area	40.7	38.7	38.4	42.4	40.9	37.8	38.8	40.8	
Urban Formal	41.8	45.2	41.5	38.2	42.1	46.8	48.0	45.4	
Urban Informal	5.8	1.8	5.5	6.1	5.2	5.8	6.7	6.2	
**Smoking [%]**									
Current Smoker	23.9	47.3	46.1	31.6	18.3	13.5	8.2	9.9	< 0.001
Never Smoker	71.4	46.1	49.1	63.4	77.4	81.5	87.3	87.4	
Past Smoker	4.7	6.6	4.8	5.0	4.3	5.0	4.5	2.7	
Missing (N)	23	1	2	7	8	3	1	1	
**Alcohol use**									
Lifetime abstainer	62.3	47.6	45.0	55.9	66.1	70.3	76.7	75.8	< 0.001
Past drinker	11.0	13.1	11.3	12.1	10.2	10.0	8.7	11.7	
Occasional Drinker	13.1	14.3	17.9	14.7	12.7	11.0	9.2	8.7	
Regular drinker	13.6	25.0	25.8	17.3	11.1	8.8	5.4	3.8	
Missing (N)	19	0	2	10	6	0	1	0	
**Physical exercise**									
Never	74.3	78.0	74.8	70.2	74.5	75.9	82.8	87.4	< 0.001
< once a week	5.4	4.8	5.7	5.4	5.3	6.2	5.2	3.1	
Once a week	4.8	4.2	3.7	5.4	5.4	4.2	3.4	3.3	
Twice a week	5.2	6.0	5.4	6.3	5.0	4.5	3.1	3.3	
> twice a week	10.2	7.1	10.4	12.7	9.9	9.2	5.5	2.9	
Missing (N)	44	0	5	18	11	5	2	3	
**Past diagnoses [%]**									
Tuberculosis	5.4	17.3	13.6	6.7	3.4	2.3	3.1	3.5	< 0.001
Hypertension	9.0	12.5	13.4	11.2	18.8	28.2	35.1	42.4	< 0.001
Diabetes	4.7	3.0	1.3	2.4	4.8	7.7	10.6	10.9	< 0.001
Stroke	1.1	1.8	1.9	0.9	0.8	1.0	1.7	2.0	0.02
Asthma	3.9	10.1	4.6	3.3	3.6	4.2	4.9	5.3	< 0.001
Heart conditions	3.6	4.2	2.8	2.7	3.4	4.5	5.8	7.1	< 0.001
Cancer	0.7	0.0	0.5	0.6	0.6	0.9	1.5	0.5	0.08
**Self-reported health [%]**									
Excellent	25.8	15.0	21.7	29.5	27.2	22.0	19.2	17.1	< 0.001
Very Good	23.8	21.0	21.0	24.6	24.6	23.7	22.5	18.7	
Good	25.4	23.4	25.7	24.0	26.6	26.0	27.5	25.9	
Fair	15.6	20.4	18.6	13.3	14.3	18.1	20.0	22.4	
Poor	9.5	20.4	13.0	8.7	7.3	10.2	10.8	16.0	
Missing (N)	59	1	3	27	13	6	5	4	

IQR = Interquartile Range; N = Number of records; p = *p*-value (null hypothesis of no difference across BMI groups).

The absolute risk of death among participants varied substantially across BMI categories. The age-standardized death rates varied from a maximum of 5161 deaths per 100,000 person-year among subjects with extremely low BMI (BMI <16 Kg/m^2^) to a minimum of 1300 per 100,000 person-year among subjects in the 30–35 Kg/m^2^ BMI range (Table 2).

**Table 2:** Crude and age-adjusted mortality rates according to Body Mass Index categories.

			Mortality rate [per 10^5^ person-year]	
BMI [kg/m^2^]	Deaths	Person-years	Crude	Age-adjusted⁎
**All sample**	1741	83,077	2096	2116
**< 16**	58	1060	5472	5161
**[16,18.5)**	180	4371	4118	4601
**[18.5,25)**	748	33,790	2214	2555
**[25,30)**	379	19,934	1901	1778
**[30,35)**	196	12,977	1510	1300
**[35,40)**	102	6585	1549	1568
**≥ 40**	78	4359	1789	1527

BMI = Body Mass Index.

⁎ Age-adjusted mortality calculated using age-specific mortality rates and the age distribution of the South African Population in 2008.

Participants excluded for lack of BMI measurements were slightly older, more frequently males, living in urban areas, of higher socioeconomic status and more often smokers and drinkers. Participants LTFU were also more often males, living in urban areas, of higher socioeconomic status and more frequently smokers and drinkers, but slightly younger. They also self-reported better overall health. In both analyses, no major differences were observed regarding patterns of chronic diseases (Supplementary Tables S1 and S2).

### Association between BMI and mortality

**Fig. 1:**
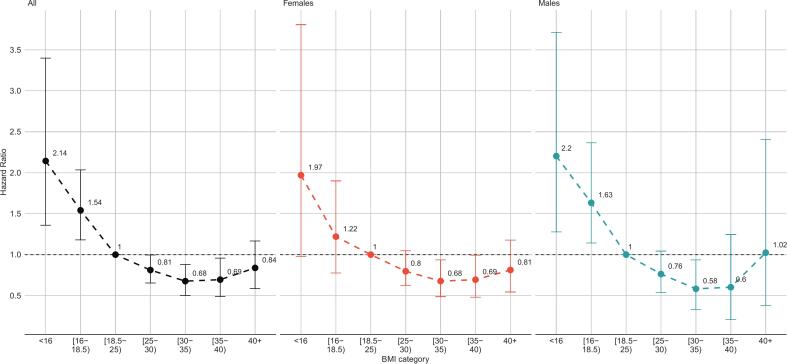
Hazard ratios and 95% confidence intervals for all-cause mortality, according to Body Mass Index and sex. South African population 20 years and older.

In the model estimated on the full sample with complete data on covariates (*N* = 10,814), HRs for all-cause death were minimal, at 0.68 (95% CI: 0.50–0.88), in the BMI range 30–35 kg/m^2^ and significantly lower than 1 also in the range 25–30 kg/m^2^ and 35–40 kg/m^2^. At the other extreme, BMI < 18.5 kg/m^2^ was associated with a significantly increased risk of death, with a maximum HR of 2.14 (1.36–3.4) in the <16 kg/m^2^ category. The pattern was repeated in the sex-specific analyses (Fig. 1).

BMI explained 2.8% of the observed variation in mortality risk among males (Royston's R^2^ = 2.8%, 95% CI: 1.1%–6.1%) vs. 1.5% (0.6%–3.7%) among females. In the age-specific analyses, the proportion of explained variation was 4.7% (2.2%–10%) among the under 40, 4.9 % (2.3%–10%) in the 40–60 year age group and 0.9% (0.5%–4.2%) in the older age group. The overall patterns suggest a weaker association between BMI and mortality among women compared to men and in the older age groups compared to the younger, albeit inter-group differences were not statistically significant.

**Fig. 2:**
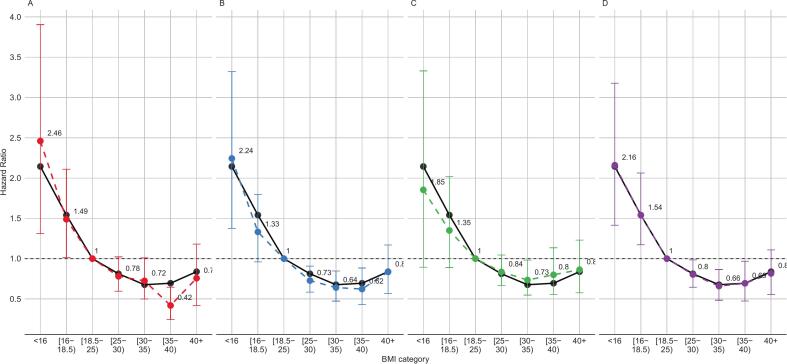
Hazard ratios and 95 % confidence intervals for all-cause mortality, according to Body Mass Index. South African population 20 years and older. Estimated excluding subjects with chronic diseases (Panel A, *N* = 7648), excluding deaths in the first 2 years of follow-up (Panel B, *N* = 10,539), excluding past and present smokers (Panel C, *N* = 7725), excluding accidental deaths (Panel D, N = 10,714). Black solid lines are full-sample estimates as per Fig. 1. Excluding subjects LTFU or adjusting the analyses using IPCW resulted in a modest decrease of the HR for low BMI values, and virtually no change above the reference category (see Supplementary Figs. S4 and S5). Repeating the analyses with multiple-imputed missing values on covariates produced negligible changes in the estimates (Supplementary Fig. S6).

The patterns of association between BMI and all-cause mortality become slightly more apparent, with a potential shift of the minimum risk towards higher BMI values, when excluding subjects with pre-existing conditions and those who died during the first two years of follow-up, thus supporting the existence of partial confounding by pre-existing conditions in the full-sample model. Excluding smokers reduces the HR for low values of BMI and modestly increases it for higher values. Excluding accidental deaths produced negligible changes in the estimates. (Fig. 2).

A summary of the estimated HRs across the different models is presented in Table 3. Coefficient estimates for the model fitted on the full sample and separately for men and women are presented in Supplementary Tables S3, S4 and S5.

**Table 3:** Hazard ratios and 95 % confidence intervals for all-cause mortality, according to Body Mass Index. South African population 20 years and older.

	Body Mass Index [kg/m^2^]						
	**< 16**	**[16,18.5)**	**[18.5,25)**	**[25,30)**	**[30,35)**	**[35,40)**	**≥ 40**
**All subjects**							
No of deaths	58	180	748	379	196	102	78
Multivariate HR^a^ (95% CI)	2.14 (1.36–3.4)	1.54 (1.18–2.03)	1 (ref)	0.81 (0.65–1)	0.68 (0.5–0.88)	0.69 (0.49–0.96)	0.84 (0.59–1.17)
Multivariate HR^a,^ IPCW (95% CI)	2.02 (1.36–2.94)	1.48 (1.13–1.92)	1 (ref)	0.81 (0.66–0.99)	0.67 (0.49–0.89)	0.69 (0.48–0.94)	0.82 (0.57–1.14)
**All males**							
No of deaths	33	122	433	146	46	16	11
Multivariate HR^b^ (95% CI)	2.20 (1.27–3.71)	1.63 (1.14–2.37)	1 (ref)	0.76 (0.54–1.04)	0.58 (0.33–0.94)	0.60 (0.21–1.25)	1.02 (0.38–2.41)
**All females**							
No of deaths	25	58	315	233	150	86	67
Multivariate HR^b^ (95% CI)	1.97 (0.98–3.81)	1.22 (0.78–1.9)	1 (ref)	0.8 (0.62–1.05)	0.68 (0.49–0.94)	0.69 (0.48–0.99)	0.81 (0.54–1.18)
**Excluding past and current smokers**							
No of deaths	20	69	428	269	163	86	66
Multivariate HR^a^ (95% CI)	1.85 (0.89–3.33)	1.35 (0.89–2.02)	1 (ref)	0.84 (0.67–1.04)	0.73 (0.55–0.98)	0.80 (0.55–1.14)	0.86 (0.58–1.23)
**Excluding chronic diseases**							
No of deaths	27	85	452	200	90	34	28
Multivariate HR^a^ (95% CI)	2.46 (1.31–3.9)	1.49 (1.02–2.11)	1 (ref)	0.78 (0.59–1.02)	0.72 (0.5–1.01)	0.42 (0.24–0.64)	0.76 (0.42–1.18)
**Excluding death in the first 2 years**							
No of deaths	48	135	628	310	172	84	68
Multivariate HR^a^	2.24 (1.38–3.32)	1.33 (0.96–1.8)	1 (ref)	0.73 (0.58–0.91)	0.64 (0.47–0.85)	0.62 (0.43–0.88)	0.84 (0.57–1.17)
**Excluding accidental deaths**							
No of deaths	54	161	690	353	188	98	75
Multivariate HR^a^ (95% CI)	2.16 (1.42–3.18)	1.54 (1.17–2.06)	1 (ref)	0.80 (0.65–0.98)	0.66 (0.48–0.86)	0.66 (0.47–0.97)	0.82 (0.55–1.11)

HR = Hazard Ratio; IPCW = Inverse Probability of Censoring Weighting.

a Adjusted for age, sex, population group, marital status, household income quintile, physical exercise, alcohol use, smoking status.

b Adjusted for age, population group, marital status, household income quintile, physical exercise, alcohol use, smoking status.

The model-predicted absolute risk of dying in the following years varied between 5.5% and 16% at 5 years, between 11% and 30% at 10 years, and between 18% and 41% at 15 years (supplementary fig. S8).

## Discussion

Consistent with major studies conducted in high-income countries (HICs), in this nationally representative South African cohort observed over 10 years, a U-shaped relationship between BMI and all-cause mortality – stronger in men and at younger ages – was found. However, in contrast with those studies – but in substantial agreement with the results of the recent work conducted by Manne-Goehler and colleagues on another population-based South African cohort [37] – mortality was lower among subjects with a BMI in the range 30–40 kg/m^2^ than among those with a BMI between 18.5 and 25 kg/m^2^. The minimum risk of death was observed in the BMI range 30–35 kg/m^2^, corresponding to Obesity Class I in the WHO classification. Similar relationships were found among women and men, with larger variations in risk per unit change of BMI observed in the latter. The relationship persisted after restricting the analyses to never smokers, excluding subjects with pre-existing conditions or who died in the first two years of follow-up, or considering only deaths for natural causes.

A combination of ethnicity-related biological factors, survival advantages in environments with a high burden of infectious diseases, dietary habits, and cultural norms may contribute to explaining these findings when interpreted in the specific context of the South African population.

‘Shifted’ ranges of minimum risk compared to the WHO recommendations (18.5 ≤ BMI < 25 kg/m^2^) have been observed for specific populations, coherently with the established evidence that the relationship between anthropometry and adiposity tends to differ by ethnic background [[54], [55], [56], [57]]. A case in point is the generally accepted evidence that South Asian populations experience the same risk of a host of health outcomes (including diabetes and cardiovascular disease) at BMI levels that are lower than non-Asian [54,55,58,59], leading the WHO to recommend lower cutpoints (BMI >22.5 kg/m^2^) for the definition of excess risk related to high BMI [60]. The population analysed in this study is prevalently of Black racial ascription (79 % of the sample self-categorises in the ‘Black African’ population group) and various studies have produced evidence of a weaker relationship between higher values of BMI and excess mortality among Black subjects, with minimum risk of death observed in the overweight or, sometimes, obese range according to the WHO definition, with relatively mild increases for higher values [18,23,[25], [26], [27],^61^,^62^]. A biologically plausible finding, given the evidence that (1) BMI overestimates body fat (and, especially, visceral fat) in African-American vs Caucasian, (2) the slope of the linear relationship between BMI and visceral fat is lower in African Americans than among Whites and Hispanic-Americans, and (3) black men and, more so, black women, have higher proportion of skeletal and muscle mass (and, consequently, lower proportion of fat mass) compared to white subjects [[63], [64], [65], [66]]. These observations – congruent with the results of this study – have led various authors to question the adequacy of the standard BMI cutpoints to define excess risk in Black populations and propose moving towards varying thresholds according to ethnicity [63,64,67,68].

.Additional adipose tissue may provide survival advantages during illness or treatment for severe infectious diseases, especially in resource-scarce environments. A consideration highly relevant in the South African context, characterised by a high burden of infectious diseases, including HIV/AIDS and tuberculosis [69,70].

Studies in HICs have repeatedly found an inverse association between various indices of diet quality and BMI/obesity risk [71,72]. However evidence from LMICs in epidemiological and dietary transition is contradictory. Higher values of two commonly used indices of diet quality (Healthy Eating Index 2015, HEI-2015, and Diet Quality Index International, DQI—I) have been found to be associated with a higher risk of obesity in the Iranian population [73], and, in South Africa, BMI in the obese range was associated with increased food diversity at the household level and, particularly, with increased consumption of pulses, fish, vegetables and fruit [74], which are known protective factors for cardiovascular diseases and other major causes of premature death [75,76]. Results of a systematic review on socioeconomic determinants of dietary patterns in LMICs suggest socioeconomic inequalities as plausible drivers of this apparently direct association between dietary quality and obesity risk, showing how high SES or living in urban areas is associated with overall healthier dietary patterns, but – differently to findings in HICs – also with higher energy, cholesterol, and saturated fat intakes [77]. A results congruent with the higher prevalence of obesity among subjects with high SES found in South Africa.

Finally, obese individuals in many sectors of the South African population may also benefit from cultural perceptions that associate higher body weight with health, wealth, and social status, potentially reducing psychosocial stress compared to contexts where obesity is stigmatised [78,79]. Various studies have linked stress levels to increased mortality, both through direct effects on inflammatory processes and indirect effects mediated by health behaviours, mental health status and healthcare practice such as medication adherence for chronic conditions [[80], [81], [82]].

In summary, the results of this study suggest that the standard BMI cutpoints may not adequately reflect excess risk in the South African population (and potentially other Black populations in LMICs), with both clinical and public health implications, in terms of diagnosis and management of diseases associated with excessive adiposity and definition of adequate targets for policy and preventive interventions. The relevance of the latter considerations in the South African population is highlighted by the large variations in the absolute risk of death across BMI categories estimated in this study. However, the amount of data collected in LMICs, especially in sub-Saharan Africa, is still limited and, moreover, substantial evidence exists that the relationship between BMI and cardiometabolic morbidity (as opposed to mortality) is not favourable to Black ethnic groups. Among other findings, studies in the UK have found an increased risk of type 2 diabetes in Black overweight and obese adults compared to their White counterparts (despite significantly lower mortality in the latter) [58,67]. In South Africa, cohort studies have found higher insulin resistance in Black women than BMI-matched white women [68], and that lower BMI cutpoints (as low as 22 kg/m^2^ for men) have better predictive power for cardiometabolic morbidity than the international standards [83]. Similarly, a study in Ethiopia found that optimal BMI cutpoints for markers of metabolic syndrome are lower than the international standards [84]. Secondary analyses in this study (see supplementary Tables S6 and S7) also confirm the discrepancy between the relationship of BMI with mortality and morbidity, and found a significantly higher risk of developing diabetes and hypertension among subjects with BMI above the standard cutoff of 25 kg/m^2^.

Caution is, therefore, warranted. More robust evidence is required before recommending changes in the current thresholds, and additional research is needed to confirm currently sparse findings.

### Strengths and limitations

Strengths of this study are the prospective design, the use of multiple measurements of weight and height (as opposed to self-report data) for the calculation of BMI, and the large sample representative at the country level. The analyses are adjusted for a number of potentially important confounders (including age at baseline, sex, smoking, alcohol consumption, physical activity, marital status, and household income quintile as an indicator of socioeconomic status) and include model checking and sensitivity analyses to assess the robustness of the estimates to violations of model assumptions regarding the absence of confounding by reverse causality in the relationship between BMI and pre-existing diseases, the shape of the baseline distribution, the proportional hazard assumption and the randomness of the censoring due to loss to follow-up.

Various limitations need to be acknowledged.

First, the proportion of participants LTFU is far from negligible, with evidence of statistically significant differences with individuals with known vital status. Proportions of this order of magnitude or greater are common in this type of study, and the sensitivity analyses do not suggest a major effect of this violation of the uninformative censoring assumption on the hazard ratio estimates, which, however, cannot be excluded.

Second, the study tried to address major sources of confounding in the observed association between BMI and mortality through statistical adjustment and sub-group analyses, which have provided reassuring results. However, the limitation of the available data leaves ample room for residual confounding. The limited quality of self-report of chronic diseases, likely affected by significant underreporting, could have reduced the effectiveness of the adjustment for reverse causality, and potentially biased downwards the hazard ratios for high values of BMI. The absence of data on HIV status, highly prevalent in the South African population and known cause of weight loss, is of particular concern, only partly mitigated by the results of the exclusion of individuals with diagnosis of tuberculosis, known to be particularly common among HIV positive individuals [85]. It is plausible that adequate adjustment for this condition would have moved the nadir of the BMI-mortality relationships towards lower BMI values, as confirmed by studies that analysed separately HIV-positive and HIV-negative subjects, which, however, found relatively modest differences [37].

Third, in the South African context of high inequality and weak social security nets, the confounding effect of SES (directly associated with BMI and inversely associated with mortality through better access to resources in general and healthcare in particular) might be stronger than in HIC settings [[86], [87], [88]]. Adjustment for income quintile as a proxy for SES might not have sufficiently addressed this potential source of bias, thus partly justifying the discrepancies of our findings with studies in other settings.

Finally, various studies have found that the length of the follow-up period affects the shape of the relationship between BMI and mortality. In particular, studies with longer follow-up tend to find the nadir of all-cause mortality at lower values of BMI [2], as an indication of biologically plausible differences between long-term and short-term effects of BMI [89,90], and, in some cases, the results of residual dilution bias, which tends to be more pronounced with longer follow-up periods [91]. Longer follow-up times for our cohort might lead to left-shifted nadirs for the BMI-mortality relationship.

## Conclusions

The results of this study suggest that, in the South African adult population, BMI in the overweight or mild obesity range according to international definitions is associated with a reduced risk of all-cause mortality compared to the”healthy weight” range.

These findings support the call by various researchers for a more individualised and flexible approach to the clinical use of standard cutpoints to define ‘healthy’ ranges of BMI, taking into account, among other factors, racial ascription, complementary indicators of adiposity and comorbidities [92,93].

Large and methodologically sound studies – including longer follow-up periods, and accurate measurements of comorbidities and causes of death – are needed to corroborate these results, provide a sound characterisation of the relationship between BMI and mortality in LMICs and sub-Saharan Africa in particular, and improve our understanding of the complex interplay of factors that shape it.

## CRediT authorship contribution statement

**Annibale Cois:** Writing – review & editing, Writing – original draft, Methodology, Formal analysis, Data curation, Conceptualization.

## Declaration of competing interest

The author reports no potential conflicts of interest, including specific financial interests, relevant to the subject of this manuscript.

## Data Availability

The anonymised microdata of the NIDS survey are available for research purposes from the Datafirst Open Data Portal (https://www.datafirst.uct.ac.za/dataportal). This study used data from NIDS wave 1dataset v.7.0, wave 2 v.4.0, wave 3 v.3.0, wave 4 v.2.0 and wave 5 v.1.0.

## Appendix A Supplementary data

Supplementary material 1: additional methods & results Image 1

Supplementary material 2: R code Image 2
