# Supplementary material for: Body mass index and mortality in a nationally representative cohort of south African adults
Source: Glob Epidemiol. 2025 Sep 26;10:100220. doi: 10.1016/j.gloepi.2025.100220 (PMC12519294; doi:10.1016/j.gloepi.2025.100220)
Supplement: Supplementary file 1 — Supplementary material 1: additional methods & results [file mmc1.pdf]

Body Mass Index and all-cause mortality in a nationally representative cohort of South African adults

Annibale Cois[1]

[1] Burden of Disease Research Unit, South African Medical Research Council & Division of Epidemiology & Biostatistics, School of Public Health, University of Cape Town

Supplementary Material 1: additional methods & results

Content

1. Additional methods 2
 1.1. Model specification and checking 2
 1.2. Weighting and adjustment of uncertainty estimates 3
2. Additional results 3
 1.3. Exclusions and sample characteristics 3
 1.4. Model coefficients 6
 1.5. Sensitivity analysis for LTFU 9
 1.6. Sensitivity analysis for exclusion of subjects with missing data on covariates 10
 1.7. Incidence of diabetes and hypertension 10
 1.8. Age-specific associations 11
 1.9. Continuous association between BMI and mortality 11
 1.10. Absolute risk estimates 12
3. References 12

1. Additional methods

1.1. Model specification and checking

The functions depicted in Figure S1 for each category X of Body Mass Index are transformations of the model-estimate of the survival function S(t|X), with the overall mean subtracted. The *diag_covar* function from the R package *icenReg* was used to draw the graph. See [1] for details.

The approximately parallel lines (net of stochastic noise) do not suggest major departures from the proportional hazard assumption underlying the statistical model.

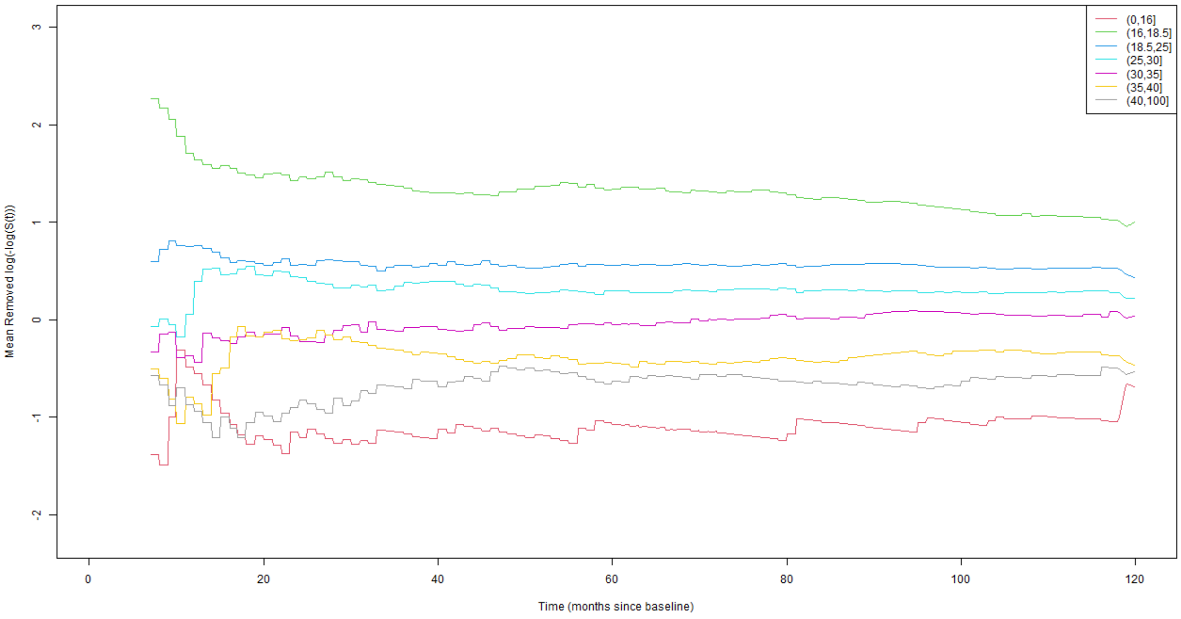

Supplementary Figure S1. Transformed survival function according to Body Mass Index categories

Figure S2 compares the Weibull baseline survival distribution assumed by the parametric model used in this study (____) with the baseline distribution recovered non-parametrically by fitting a cox-PH model (____). The substantial overlap of the two curves suggests the adequacy of the model assumption of a Weibull distribution for the baseline survival.

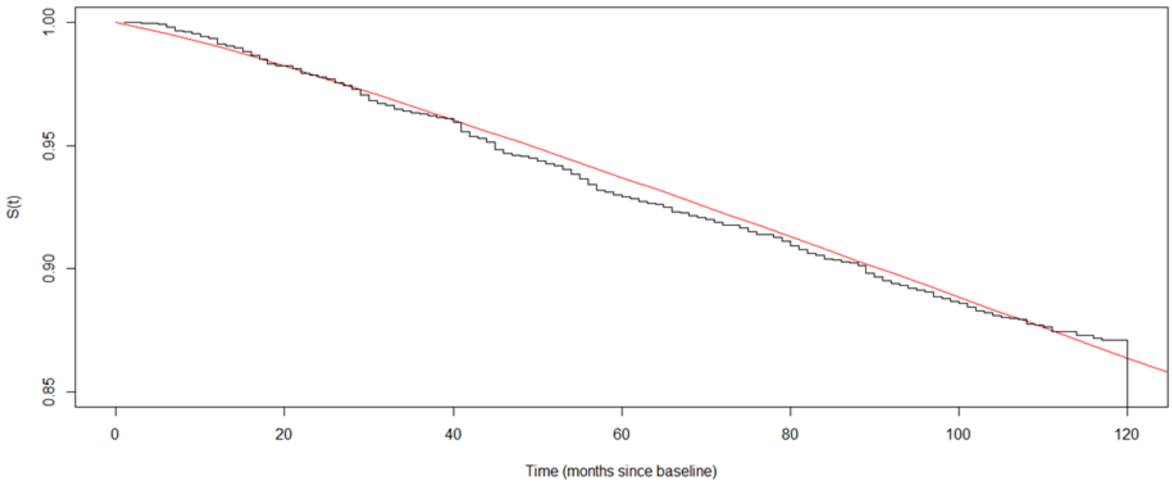

Supplementary Figure S2. Baseline survival distribution

1.2. Weighting and adjustment of uncertainty estimates

The estimates shown in the manuscript account for the complex strategy used to select the sample in the NIDS survey, including clustering and stratification.

Calibrated longitudinal sampling weights provided with the survey dataset[2] were rescaled to sum to the total sample size and used in the estimation to improve the representativeness of the estimates.[1]

A bootstrap procedure was implemented to correct the confidence intervals for the clustered and stratified sampling design of the NIDS survey. The R library *surveybootstrap*[3] was used to extract 2000 bootstrap samples from the dataset, and, at each iteration, the various models were refit and the relevant point estimates extracted. The 2.5th and 97.5th percentiles of the extracted distribution of each estimate were considered as the bounds of its 95% confidence interval.

2. Additional results

1.3. Exclusions and sample characteristics

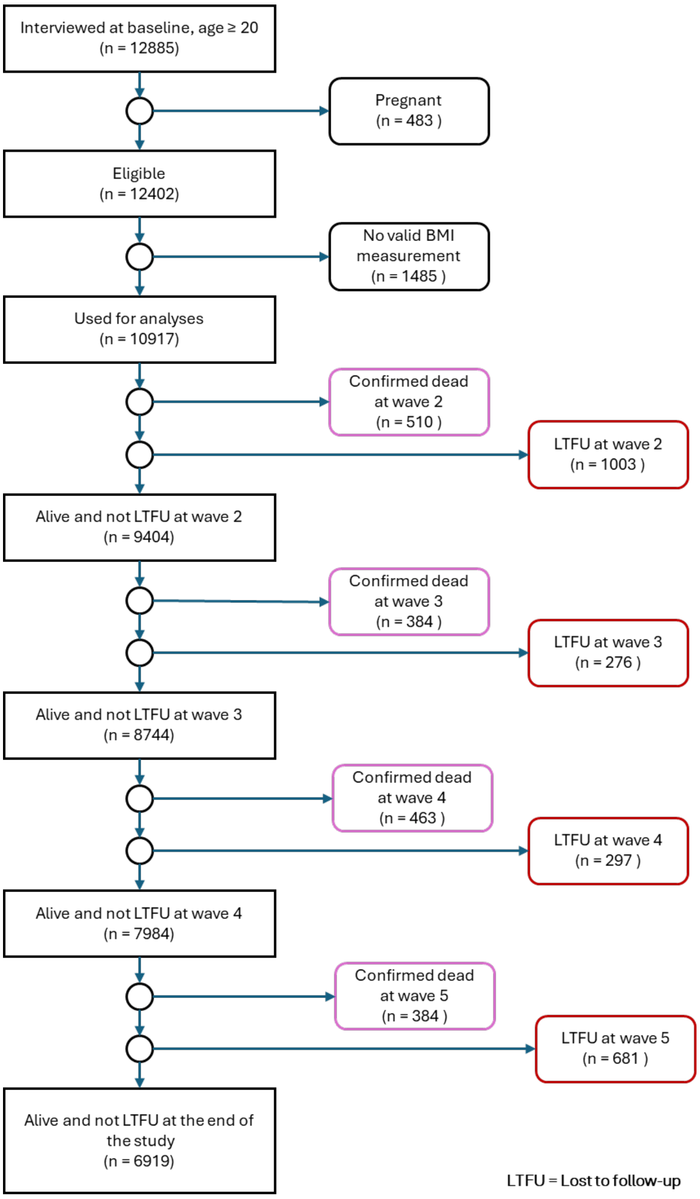

Supplementary Figure S3. Cohort selection process and evolution

Supplementary Table S1. Baseline characteristics of participants with and without valid BMI measurements.

	Valid BMI measurement		
	Yes (N=10917)	No (N=1485)	p
Sex [%]			
Female	60.7	55.4	< 0.001
Age [Years]			
Median (IQR)	40.0 (28.0-54.0)	44.0 (31.0-59.0)	
Population group [%]			
Black African	78.9	61.4	< 0.001
Coloured	14.2	20.3	
White	5.5	15.6	
Asian	1.3	2.7	
Marital status [%]			
Single	42.1	35.6	< 0.001
Married/Informal Union	44.3	46.4	
Widowed/Separated/Divorced	13.6	18.0	
Missing (N)	31	9	
Education [%]			
No school	18.8	21.3	< 0.001
Primary	31.0	25.0	
Secondary	39.9	37.1	
Tertiary	10.3	16.7	
Missing (N)	1485	260	
Employment [%]			
Not Economically Active	34.5	37.6	< 0.001
Unemployed	20.4	14.6	
Employed	45.1	47.8	
Missing	97	17	
Income Quintile [%]			
I (poorest)	18.4	13.1	< 0.001
II	19.3	17.3	
III	20.6	17.1	
IV	21.3	21.3	
V (richest)	20.4	30.7	
Geotype [%]			
Rural Formal	11.6	8.4	< 0.001
Tribal Area	40.7	22.4	
Urban Formal	41.8	61.4	
Urban Informal	5.8	7.8	
Smoking [%]			
Current Smoker	23.9	28.0	< 0.001
Never Smoker	71.4	65.6	
Past Smoker	4.7	6.5	
Missing	23	19	
Alcohol use			
Lifetime abstainer	62.3	53.3	< 0.001
Past drinker	11.0	13.8	
Occasional Drinker	13.1	15.2	
Regular drinker	13.6	17.6	
Missing	19	17	
Physical exercise			
Never	74.3	66.1	< 0.001
< once a week	5.4	8.1	
Once a week	4.8	7.3	
Twice a week	5.2	5.5	
> twice a week	10.2	13.0	
Missing (N)	44	24	
Past diagnoses [%]			
Tuberculosis	5.4	4.4	< 0.001
Hypertension	(19.0%)	21.3	0.077
Diabetes	4.7%)	5.3	0.001
Stroke	1.1	2.3	0.001
Asthma	3.9	3.2	0.241
Heart conditions	3.6	4.2	0.087
Cancer	0.7	0.9	< 0.001
Self-reported health [%]			
Excellent	25.8	26.0	0.127
Very Good	23.8	25.6	
Good	25.4	22.5	
Fair	15.6	15.6	
Poor	9.5	10.4	
Missing (N)	59	20	

IQR = Interquartile Range; N = Number of records.

p = p-value (null hypothesis of no difference between participants with and without valid BMI measurements).

Supplementary Table S2. Baseline characteristics of the study sample according to follow-up status.

	LFTU		
	Yes (N=8660)	No (N=2257)	p
**Sex [%]**			
Female	63.2	51.2	< 0.001
**Age [Years]**			
Median (IQR)	41.0 (29.0-55.0)	35.0 (26.0-48.0)	< 0.001
**Population group [%]**			
Black African	81.9	67.6	< 0.001
Coloured	14.1	14.9	
White	3.0	15.2	
Asian	1.1	2.3	
**Marital status [%]**			
Single	41.8	43.5	< 0.001
Married/Informal Union	43.6	46.8	
Widowed/Separated/Divorced	14.6	9.8	
Missing (N)	27	4	
**Education [%]**			
No school	19.8	14.8	< 0.001
Primary	32.6	24.5	
Secondary	39.5	41.7	
Tertiary	8.2	19.0	
Missing (N)	1062	423	
**Employment [%]**			
Not Economically Active	36.6	26.3	< 0.001
Unemployed	21.0	18.2	
Employed	42.4	55.5	
Missing	80	17	
**Income Quintile [%]**			
I (poorest)	20.0	12.2	< 0.001
II	20.8	13.4	
III	21.8	16.3	
IV	21.4	21.1	
V (richest)	16.0	37.0	
**Geotype [%]**			
Rural Formal	10.9	14.5	< 0.001
Tribal Area	44.6	25.9	
Urban Formal	38.9	53.2	
Urban Informal	5.6	6.4	
**Smoking [%]**			
Current Smoker	22.7	28.5	< 0.001
Never Smoker	72.9	65.5	
Past Smoker	4.4	6.0	
Missing	18	5	
**Alcohol use**			
Lifetime abstainer	65.1	51.4	< 0.001
Past drinker	10.9	11.6	
Occasional Drinker	12.0	17.7	
Regular drinker	12.1	19.3	
Missing	16	3	
**Physical exercise**			
Never	76.8	64.5	< 0.001
< once a week	5.1	6.6	
Once a week	4.3	6.8	
Twice a week	4.7	7.4	
> twice a week	9.1	14.7	
Missing (N)	32	12	
**Past diagnoses [%]**			
Tuberculosis	5.8	3.9	< 0.001
Hypertension	20.1	15.0	0.077
Diabetes	4.9	4.0	0.001
Stroke	1.2	0.4	0.001
Asthma	4.2	2.7	0.241
Heart	3.7	3.2	0.087
Cancer	0.6	0.9	< 0.001
**Self-reported health [%]**			
Excellent	24.2	31.6	< 0.001
Very Good	22.7	27.8	
Good	25.8	23.6	
Fair	16.8	11.0	
Poor	10.4	6.1	
Missing (N)	49	10	
**BMI [kg/m^2]**			
Median (IQR)	25.3 (21.5-30.9)	24.9 (21.5-29.7)	0.010

IQR = Interquartile Range; LTFU = Lost to follow-up; N = Number of records;
p = p-value (null hypothesis of no difference between LTFU and non-LTFU).

1.4. Model coefficients

Supplementary Table S3. Coefficients of the proportional hazard model: full sample

Coefficient	Estimate	Exp(Est)	Std.Error*	z-value*	p*
log_shape	0.1603	1.174	0.02541	6.306	2.86E-10
log_scale	6.424	616.8	0.0522	123.1	0.00E+00
BMICAT(0,16]	0.7625	2.144	0.1584	4.814	1.48E-06
BMICAT(16,18.5]	0.4322	1.541	0.09888	4.371	1.24E-05
BMICAT(25,30]	-0.208	0.8122	0.07166	-2.903	3.70E-03
BMICAT(30,35]	-0.3908	0.6765	0.09025	-4.33	1.49E-05
BMICAT(35,40]	-0.3642	0.6948	0.1209	-3.011	2.61E-03
BMICAT(40,100]	-0.1765	0.8382	0.1268	-1.391	1.64E-01
AGECAT5(20,25]	0.6185	1.856	0.1959	3.158	1.59E-03
AGECAT5(25,30]	1.152	3.164	0.1886	6.107	1.01E-09
AGECAT5(30,35]	1.324	3.759	0.1897	6.982	2.91E-12
AGECAT5(35,40]	1.41	4.096	0.1915	7.365	1.77E-13
AGECAT5(40,45]	1.444	4.238	0.1925	7.502	6.26E-14
AGECAT5(45,50]	1.77	5.871	0.1907	9.282	0.00E+00
AGECAT5(50,55]	1.809	6.107	0.1921	9.417	0.00E+00
AGECAT5(55,60]	2.058	7.828	0.1927	10.68	0.00E+00
AGECAT5(60,65]	2.405	11.08	0.1938	12.41	0.00E+00
AGECAT5(65,70]	2.856	17.39	0.1908	14.96	0.00E+00
AGECAT5(70,75]	2.857	17.4	0.2044	13.97	0.00E+00
AGECAT5(75,120]	3.298	27.07	0.1974	16.71	0.00E+00
RACEColoured	-0.3053	0.7369	0.1085	-2.813	4.91E-03
RACEAsian	-0.1281	0.8798	0.2135	-0.6	5.49E-01
RACEWhite	-0.03533	0.9653	0.1263	-0.2796	7.80E-01
SEXFemale	-0.2401	0.7865	0.06693	-3.588	3.33E-04
INCQ(253,414]	-0.3069	0.7358	0.08717	-3.52	4.31E-04
INCQ(414,692]	-0.1975	0.8207	0.08442	-2.34	1.93E-02
INCQ(692,1.38e+03]	-0.1108	0.8951	0.08193	-1.353	1.76E-01
INCQ(1.38e+03,9.38e+04]	-0.3801	0.6838	0.09598	-3.961	7.48E-05
FEXERLess than once a week	-0.06979	0.9326	0.1117	-0.6248	5.32E-01
FEXEROnce a week	-0.3969	0.6724	0.1336	-2.971	2.97E-03
FEXERTwice a week	-0.6505	0.5218	0.1411	-4.611	4.00E-06
FEXERThree or more times a week	-0.3554	0.7009	0.1025	-3.467	5.27E-04
ALCATPast drinker	0.1854	1.204	0.1346	1.377	1.69E-01
ALCATOccasional Drinker	-0.1408	0.8687	0.1326	-1.062	2.88E-01
ALCATRegular drinker	-0.1811	0.8343	0.1187	-1.526	1.27E-01
SMOCATNever Smoker	-0.4894	0.613	0.09901	-4.943	7.68E-07
SMOCATPast Smoker	-0.4305	0.6502	0.2298	-1.873	6.11E-02
MARSTATUSMarried/Informal Union	-0.3446	0.7085	0.06935	-4.969	6.73E-07
MARSTATUSWidowed/Separated/Divorced	-0.06367	0.9383	0.08836	-0.7206	4.71E-01
mSpline(BASELINE, knots = c(3, 6, 9), intercept = FALSE, periodic = TRUE, Boundary.knots = c(1, 12))1	-1.274	0.2797	0.5545	-2.298	2.16E-02
mSpline(BASELINE, knots = c(3, 6, 9), intercept = FALSE, periodic = TRUE, Boundary.knots = c(1, 12))2	-0.445	0.6409	0.3574	-1.245	2.13E-01
mSpline(BASELINE, knots = c(3, 6, 9), intercept = FALSE, periodic = TRUE, Boundary.knots = c(1, 12))3	-0.4941	0.6101	0.4357	-1.134	2.57E-01
ALCATPast drinker:SMOCATNever Smoker	0.1431	1.154	0.171	0.8365	4.03E-01
ALCATOccasional Drinker:SMOCATNever Smoker	0.2854	1.33	0.1711	1.668	9.54E-02
ALCATRegular drinker:SMOCATNever Smoker	0.1262	1.135	0.1733	0.7283	4.66E-01
ALCATPast drinker:SMOCATPast Smoker	0.3907	1.478	0.2952	1.323	1.86E-01
ALCATOccasional Drinker:SMOCATPast Smoker	-0.3339	0.7161	0.3919	-0.8519	3.94E-01
ALCATRegular drinker:SMOCATPast Smoker	0.4545	1.575	0.3024	1.503	1.33E-01

* Standard errors, z-values and p-values shown here are before adjustment for clustering and stratification. The confidence intervals shown and interpreted in the manuscript include adjustment by bootstrap replication (see above for details).

Supplementary Table S4. Coefficients of the proportional hazard model: males

Coefficient	Estimate	Exp(Est)	Std.Error*	z-value*	P*
log_shape	0.1801	1.197	0.03842	4.687	2.78E-06
log_scale	6.287	537.7	0.07589	82.85	0.00E+00
BMICAT(0,16]	0.7897	2.203	0.2033	3.885	1.02E-04
BMICAT(16,18.5]	0.4902	1.633	0.1286	3.811	1.39E-04
BMICAT(25,30]	-0.2702	0.7633	0.1138	-2.375	1.76E-02
BMICAT(30,35]	-0.5395	0.583	0.1783	-3.026	2.48E-03
BMICAT(35,40]	-0.507	0.6023	0.3456	-1.467	1.42E-01
BMICAT(40,100]	0.02344	1.024	0.3025	0.07749	9.38E-01
AGECAT5(20,25]	0.5251	1.691	0.441	1.191	2.34E-01
AGECAT5(25,30]	0.7954	2.215	0.4424	1.798	7.22E-02
AGECAT5(30,35]	1.21	3.354	0.4413	2.742	6.11E-03
AGECAT5(35,40]	1.464	4.324	0.4468	3.277	1.05E-03
AGECAT5(40,45]	1.112	3.04	0.4604	2.415	1.57E-02
AGECAT5(45,50]	1.805	6.078	0.453	3.984	6.77E-05
AGECAT5(50,55]	1.946	7	0.457	4.258	2.07E-05
AGECAT5(55,60]	1.97	7.172	0.4643	4.244	2.20E-05
AGECAT5(60,65]	2.343	10.41	0.4631	5.059	4.22E-07
AGECAT5(65,70]	2.958	19.25	0.458	6.458	1.06E-10
AGECAT5(70,75]	3.056	21.25	0.474	6.447	1.14E-10
AGECAT5(75,120]	3.624	37.48	0.4651	7.791	6.66E-15
RACEColoured	-0.1517	0.8592	0.1633	-0.9294	3.53E-01
RACEAsian	-0.1988	0.8197	0.3565	-0.5578	5.77E-01
RACEWhite	0.01984	1.02	0.1823	0.1088	9.13E-01
SEXFemale	-0.4179	0.6584	0.1461	-2.86	4.24E-03
INCQ(253,414]	-0.3134	0.7309	0.1379	-2.273	2.31E-02
INCQ(414,692]	-0.3686	0.6917	0.1312	-2.809	4.98E-03
INCQ(692,1.38e+03]	-0.2746	0.7598	0.1415	-1.94	5.23E-02
INCQ(1.38e+03,9.38e+04]	-0.05059	0.9507	0.1557	-0.3249	7.45E-01
FEXERLess than once a week	-0.6462	0.524	0.2044	-3.162	1.57E-03
FEXEROnce a week	-0.429	0.6512	0.1806	-2.376	1.75E-02
FEXERTwice a week	-0.4629	0.6294	0.1379	-3.356	7.89E-04
FEXERThree or more times a week	0.04564	1.047	0.1932	0.2363	8.13E-01
ALCATPast drinker	-0.1593	0.8528	0.1746	-0.912	3.62E-01
ALCATOccasional Drinker	-0.1612	0.8512	0.1583	-1.018	3.09E-01
ALCATRegular drinker	-0.5436	0.5807	0.1514	-3.59	3.31E-04
SMOCATNever Smoker	-0.5493	0.5773	0.4581	-1.199	2.30E-01
SMOCATPast Smoker	-0.4179	0.6584	0.1133	-3.688	2.26E-04
MARSTATUSMarried/Informal Union	-0.1607	0.8516	0.1729	-0.9291	3.53E-01
MARSTATUSWidowed/Separated/Divorced	-3.112	0.04451	1.605	-1.939	5.25E-02
mSpline(BASELINE, knots = c(3, 6, 9), intercept = FALSE, periodic = TRUE, Boundary.knots = c(1, 12))1	-0.2396	0.7869	0.6694	-0.3579	7.20E-01
mSpline(BASELINE, knots = c(3, 6, 9), intercept = FALSE, periodic = TRUE, Boundary.knots = c(1, 12))2	-0.8469	0.4287	1.041	-0.8138	4.16E-01
mSpline(BASELINE, knots = c(3, 6, 9), intercept = FALSE, periodic = TRUE, Boundary.knots = c(1, 12))3	0.6281	1.874	0.2583	2.431	1.50E-02
ALCATPast drinker:SMOCATNever Smoker	0.4019	1.495	0.2472	1.626	1.04E-01
ALCATOccasional Drinker:SMOCATNever Smoker	0.205	1.228	0.2447	0.8377	4.02E-01
ALCATRegular drinker:SMOCATNever Smoker	0.8231	2.278	0.5276	1.56	1.19E-01
ALCATPast drinker:SMOCATPast Smoker	-0.4514	0.6367	0.7324	-0.6164	5.38E-01
ALCATOccasional Drinker:SMOCATPast Smoker	-0.02871	0.9717	0.5618	-0.0511	9.59E-01
ALCATRegular drinker:SMOCATPast Smoker	0.1801	1.197	0.03842	4.687	2.78E-06

* Standard errors, z-values and p-values shown here are before adjustment for clustering and stratification. The confidence intervals shown and interpreted in the manuscript include adjustment by bootstrap replication (see above for details).

Supplementary Table S5. Coefficients of the proportional hazard model: females

Coefficient	Estimate	Exp(Est)	Std.Error*	z-value*	P*
log_shape	0.1534	1.166	0.03431	4.472	7.74E-06
log_scale	6.548	698.1	0.0745	87.9	0.00E+00
BMICAT(0,16]	0.678	1.97	0.2948	2.3	2.15E-02
BMICAT(16,18.5]	0.1989	1.22	0.1743	1.141	2.54E-01
BMICAT(25,30]	-0.2268	0.7971	0.09542	-2.377	1.75E-02
BMICAT(30,35]	-0.3899	0.6771	0.1067	-3.653	2.59E-04
BMICAT(35,40]	-0.365	0.6942	0.133	-2.745	6.05E-03
BMICAT(40,100]	-0.2076	0.8125	0.1412	-1.47	1.41E-01
AGECAT5(20,25]	0.7356	2.087	0.3565	2.064	3.90E-02
AGECAT5(25,30]	1.477	4.379	0.3489	4.233	2.31E-05
AGECAT5(30,35]	1.4	4.055	0.3516	3.982	6.83E-05
AGECAT5(35,40]	1.344	3.833	0.3556	3.778	1.58E-04
AGECAT5(40,45]	1.761	5.817	0.3539	4.976	6.49E-07
AGECAT5(45,50]	1.67	5.312	0.3594	4.646	3.39E-06
AGECAT5(50,55]	1.662	5.269	0.3569	4.657	3.21E-06
AGECAT5(55,60]	2.153	8.612	0.3553	6.06	1.36E-09
AGECAT5(60,65]	2.452	11.61	0.3546	6.913	4.74E-12
AGECAT5(65,70]	2.81	16.61	0.3567	7.877	3.33E-15
AGECAT5(70,75]	2.677	14.54	0.3637	7.36	1.84E-13
AGECAT5(75,120]	3.029	20.68	0.3605	8.403	0.00E+00
RACEColoured	-0.5977	0.5501	0.1588	-3.763	1.68E-04
RACEAsian	0.07609	1.079	0.2789	0.2728	7.85E-01
RACEWhite	-0.2133	0.8079	0.2029	-1.051	2.93E-01
SEXFemale	-0.249	0.7796	0.1104	-2.256	2.41E-02
INCQ(253,414]	-0.1348	0.8739	0.1099	-1.226	2.20E-01
INCQ(414,692]	0.1563	1.169	0.1081	1.446	1.48E-01
INCQ(692,1.38e+03]	-0.531	0.588	0.1485	-3.577	3.48E-04
INCQ(1.38e+03,9.38e+04]	0.01652	1.017	0.1763	0.09373	9.25E-01
FEXERLess than once a week	-0.0284	0.972	0.1857	-0.1529	8.79E-01
FEXEROnce a week	-0.9691	0.3794	0.2732	-3.548	3.89E-04
FEXERTwice a week	-0.1532	0.858	0.1737	-0.8819	3.78E-01
FEXERThree or more times a week	0.5992	1.821	0.2467	2.429	1.51E-02
ALCATPast drinker	0.1042	1.11	0.2808	0.371	7.11E-01
ALCATOccasional Drinker	-0.1709	0.8429	0.2658	-0.6432	5.20E-01
ALCATRegular drinker	-0.5182	0.5956	0.1649	-3.142	1.68E-03
SMOCATNever Smoker	-0.2527	0.7767	0.3744	-0.6751	5.00E-01
SMOCATPast Smoker	-0.3382	0.7131	0.09459	-3.575	3.50E-04
MARSTATUSMarried/Informal Union	0.1458	1.157	0.1075	1.357	1.75E-01
MARSTATUSWidowed/Separated/Divorced	0.5815	1.789	1.075	0.5407	5.89E-01
mSpline(BASELINE, knots = c(3, 6, 9), intercept = FALSE, periodic = TRUE, Boundary.knots = c(1, 12))1	-0.5091	0.6011	0.55	-0.9256	3.55E-01
mSpline(BASELINE, knots = c(3, 6, 9), intercept = FALSE, periodic = TRUE, Boundary.knots = c(1, 12))2	-0.0644	0.9376	0.7407	-0.08695	9.31E-01
mSpline(BASELINE, knots = c(3, 6, 9), intercept = FALSE, periodic = TRUE, Boundary.knots = c(1, 12))3	-0.6012	0.5481	0.2985	-2.014	4.40E-02
ALCATPast drinker:SMOCATNever Smoker	0.01802	1.018	0.3221	0.05595	9.55E-01
ALCATOccasional Drinker:SMOCATNever Smoker	-0.1383	0.8709	0.3534	-0.3912	6.96E-01
ALCATRegular drinker:SMOCATNever Smoker	-0.8583	0.4239	0.5969	-1.438	1.51E-01
ALCATPast drinker:SMOCATPast Smoker	0.1907	1.21	0.7786	0.245	8.07E-01
ALCATOccasional Drinker:SMOCATPast Smoker	1.758	5.799	0.5044	3.484	4.93E-04
ALCATRegular drinker:SMOCATPast Smoker	0.1534	1.166	0.03431	4.472	7.74E-06

* Standard errors, z-values and p-values shown here are before adjustment for clustering and stratification. The confidence intervals shown and interpreted in the manuscript include adjustment by bootstrap replication (see above for details).

1.5. Sensitivity analysis for LTFU

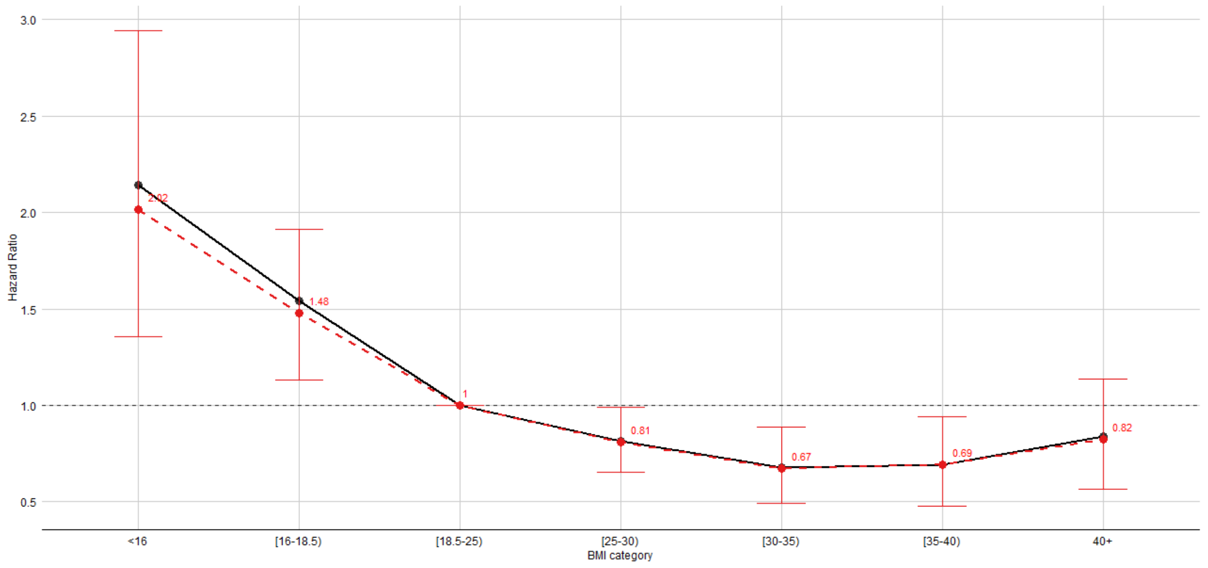

Supplementary Figure S4. Hazard ratios and 95% confidence intervals for all-cause mortality, according to Body Mass Index. Excluding participants LTFU (N = 7648),
Black solid lines are full-sample estimates as per Figure 1.

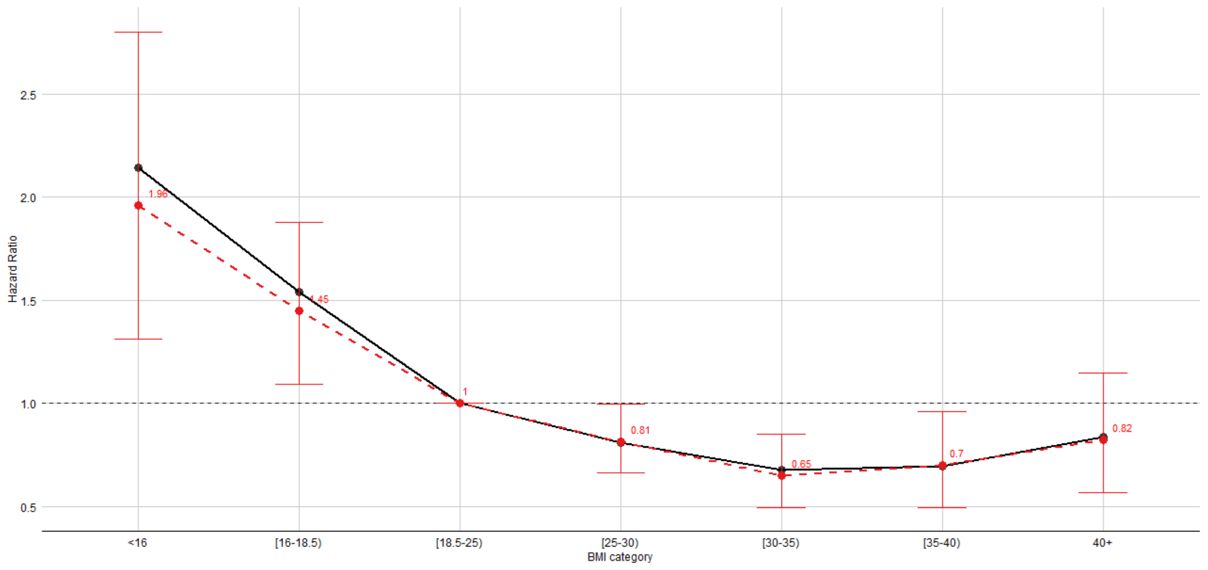

Supplementary Figure S5. Hazard ratios and 95% confidence intervals for all-cause mortality, according to Body Mass Index. Estimates adjusted by inverse probability of censoring weighting.
Black solid lines are full-sample estimates as per Figure 1.

1.6. Sensitivity analysis for exclusion of subjects with missing data on covariates

As shown in Table 1, missing data were present in some of the covariates included in the models, namely smoking status (23 missing observations), alcohol consumption (19 missing observations), marital status (31 missing observations) and frequency of physical exercise (44 missing observations). The exclusion of subjects with missing data in one or more of these variables led to a modest decrease in the sample, by 103 units (or 0.94% of the original sample). This modest proportion of missing data led to the choice of a complete data approach for the main analysis.

To assess the potential magnitude of the bias introduced by this choice, the analyses were repeated on 30 multiple imputed datasets (Multiple imputation with chained equation (R Package MICE [4]. Results combined with Rubin's rule [5]) The results do not show appreciable differences with the complete case analysis approach (Figure S6).

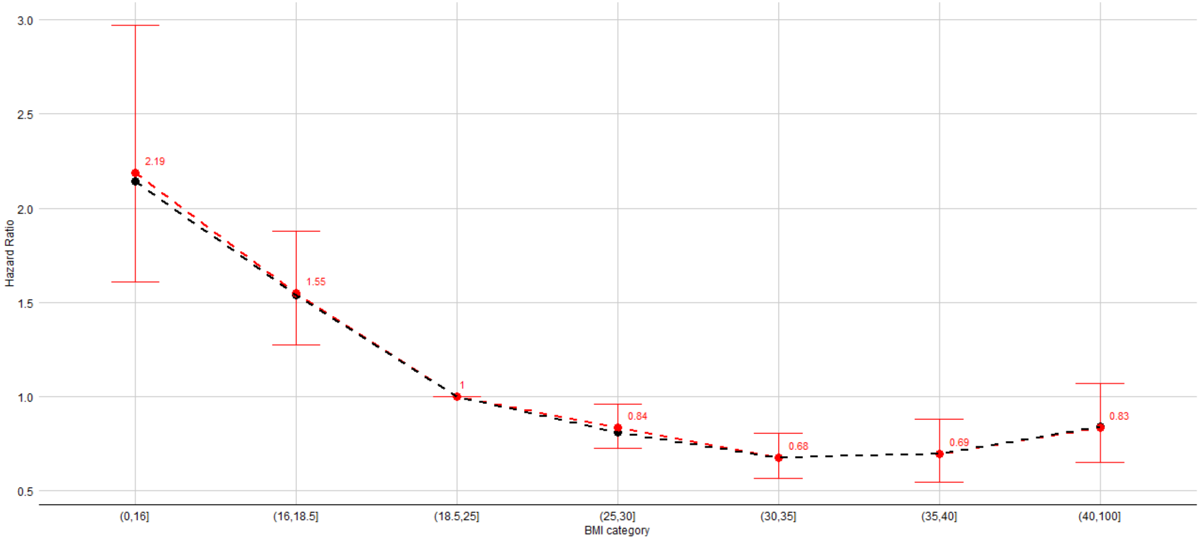

Supplementary Figure S6. Hazard ratios and 95% confidence intervals for all-cause mortality, according to Body Mass Index. Estimates including the full sample (N = 10917) with multiple imputation for missing values on covariates.
Black solid lines are full-sample estimates as per Figure 1.

1.7. Incidence of diabetes and hypertension

Supplementary Tables S6 and S7 report the age and race-adjusted relative odds of developing diabetes (S6) and hypertension (S7) during the period of follow-up among subjects disease-free at baseline. Estimates are adjusted for the survey design.

Supplementary Table S6. Relative odds of developing diabetes among subjects diabetes-free at baseline, by BMI category

BMI category	Logistic coefficient	Standard Error	OR	p
BMI < 16	-0.1118	0.5516	1.74	0.839
16 ≤ BMI < 18.5	-0.1270	0.4073	1.5	0.755
18.5 ≤ BMI < 25 (reference)	0	0	1	-
25 ≤ BMI < 30	0.5079	0.1646	1.18	0.002
30 ≤ BMI < 35	1.3274	0.1893	1.21	<0.001
35 ≤ BMI < 40	1.3598	0.2314	1.26	<0.001
BMI ≥ 40	1.4251	0.2560	1.29	<0.001

Supplementary Table S7. Relative odds of developing hypertension among subjects normotensive at baseline, by BMI category

BMI category	Logistic coefficient	Standard Error	OR	p
BMI < 16	-0.31579	0.33987	1.4	0.353
16 ≤ BMI < 18.5	-0.31570	0.22666	1.25	0.164
18.5 ≤ BMI < 25 (reference)	0	0	1	-
25 ≤ BMI < 30	0.25961	0.09943	1.1	0.009
30 ≤ BMI < 35	0.73921	0.12815	1.14	<0.001
35 ≤ BMI < 40	1.11769	0.16777	1.18	<0.001
BMI ≥ 40	0.86570	0.20091	1.22	<0.001

1.8. Age-specific associations

Supplementary Table S8. Hazard ratios and 95% confidence intervals for all-cause mortality, according to Body Mass Index. South African population 20 years and older. By age group.

	Body Mass Index [kg/m^2]						
	< 16	[16,18.5)	[18.5,25)	[25,30)	[30,35)	[35,40)	≥ 40
**< 40 years**							
No of deaths	11	62	254	78	31	17	10
Multivariate HR (95% CI)	1.8 (0.62-3.6)	2.0 (1.2-3.0)	1 (ref)	0.79 (0.55-1.1)	0.59 (0.30-0.98)	0.51 (0.23-0.92)	0.91 (0.34-1.9)
**40 to 59 years**							
No of deaths	29	64	224	120	66	42	26
Multivariate HR (95% CI)	2.6 (1.5 – 4.4)	1.6 (0.95-2.5)	1 (ref)	0.69 (0.44-1.1)	0.60 (0.34-0.96)	0.68 (0.37-1.2)	0.56 (0.27-0.99)
**60 years and over**							
No of deaths	18	54	270	181	99	43	42
Multivariate HR (95% CI)	1.8 (0.75-3.7)	1.1 (0.59-1.8)	1 (ref)	1.1 (0.80-1.5)	0.94 (0.59-1.5)	0.83 (0.48-1.4)	1.2 (0.68-2.0)

HR = Hazard Ratio. Estimates adjusted for sex, population group, marital status, household income quintile, physical exercise, alcohol use, smoking status.

1.9. Continuous association between BMI and mortality

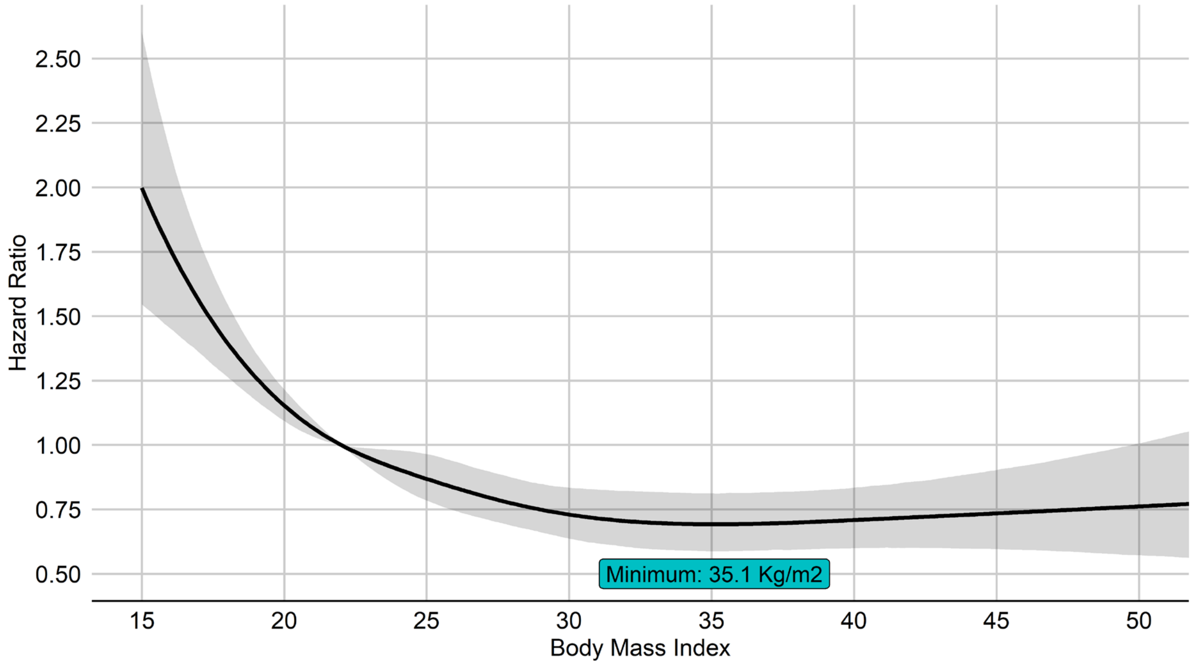

Supplementary Figure S7. Association between BMI and all-cause mortality in the South African population 20 years and older. Hazard ratio and 95% confidence interval modelled using a natural cubic spline with 6 degrees of freedom in an interval-censored survival model. Estimates adjusted for sex, population group, marital status, household income quintile, physical exercise, alcohol use, smoking status.

1.10. Absolute risk estimates

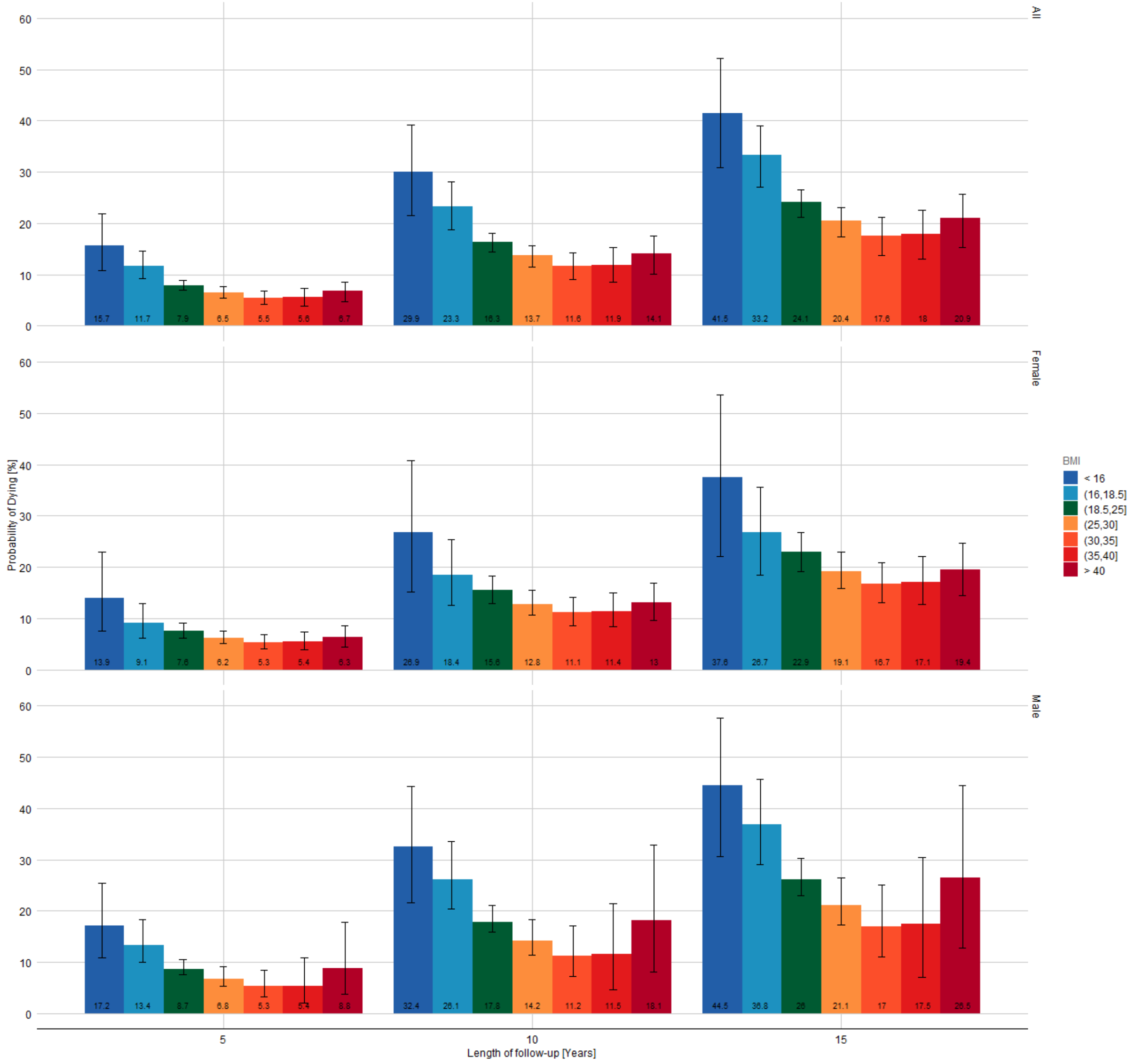

Supplementary Figure S8. Model predictions of the absolute risk of dying in the following 5, 10 and 15 years for a population with the covariate distribution of the South African adult population in 2008 and BMI in different categories.

3. References

1. Anderson-Bergman C. icenReg: Regression Models for Interval Censored Data in R. *Journal of Statistical Software*. 2017;81:1-23. doi:10.18637/jss.v081.i12

2. Branson N, Wittenberg M. Longitudinal and cross-sectional weights in the NIDS data 1-5. *Cape Town: SALDRU(NIDS Technical Paper 8)*. Published online 2018.

3. Feehan DM. *Surveybootstrap: Bootstrap with Survey Data*.; 2023. https://CRAN.R-project.org/package=surveybootstrap

4. van Buuren S, Groothuis-Oudshoorn K. {mice}: Multivariate Imputation by Chained Equations in R. *Journal of Statistical Software*. 2011;45(3):1-67.

5. Rubin DB. Multiple imputation. In: *Flexible Imputation of Missing Data, Second Edition*. Chapman and Hall/CRC; 2018:29-62.
